# Decreased sperm counts in Swedish users of oral tobacco

**DOI:** 10.1111/andr.13198

**Published:** 2022-06-08

**Authors:** Agnes Kimblad, Gustav Ollvik, Christian H Lindh, Jonatan Axelsson

**Affiliations:** ^1^ Reproductive Medicine Centre Skåne University Hospital Malmö Malmö Sweden; ^2^ Molecular Reproductive Medicine Department of Translational Medicine Lund University Malmö Sweden; ^3^ Division of Occupational and Environmental Medicine Lund University Lund Sweden

**Keywords:** nicotine, semen quality, snuff, snus, tobacco

## Abstract

**Background:**

Smoke‐free tobacco via moist oral snuff (snus) is used daily in more than 20% of Swedish men. Negative effects of cigarette smoking on sperm parameters are well documented, unlike for snuff, despite relevance also for other smoke‐free nicotine products.

**Objectives:**

We wanted to investigate whether reproductive parameters differed between users and non‐users of snuff, and whether the amount of snuff and nicotine exposure mattered.

**Materials and methods:**

Men (*n* = 613) from the general population, recruited 2000–2010, were physically examined, answered questions on smoking and snuff use, and delivered urine, blood and semen samples. Sperm concentration, total sperm count, semen volume, percent morphologically normal and progressively motile sperm, and DNA fragmentation index (by the Sperm Chromatin Structure Assay) and reproductive hormones were analysed. Nicotine exposure was measured through urinary levels of cotinine. We used general linear models, with adjustments including cigarette smoking, and for semen parameters also abstinence time.

**Results:**

After adjustments, total sperm count was 24% lower (*P* = 0.03) and testosterone 14% higher (*P* < 0.001) in 109 users of snuff than in non‐users, whereas cotinine was positively associated with testosterone and oestradiol (*P* < 0.001). Numbers of boxes of snuff used per week were associated with testosterone and FSH (*P* < 0.001).

**Discussion:**

Applied to the general population, the consumption of smoke‐free tobacco by the use of snuff was associated with a lower sperm count and a higher testosterone, for which the extent seemed to play a role.

**Conclusions:**

Independent of smoking, consumption of snuff was associated with lower total sperm count and different hormone levels. Applying these results to a reported association between sperm count and the chance of pregnancy, men who used snuff would have about a 10% lower chance of fathering a child.

## 1 INTRODUCTION

As much as 15% of couples may have fertility problems,^1^ and men's tobacco use via smoking may play a role via reduced semen parameters.^2^ Still, also oral tobacco (chewing) has been associated with a decreased semen quality,^3^ which may be relevant to the use of smokeless tobacco that has increased in recent years.^4^ Smokeless tobacco through moist oral snuff, locally called “snus”, is used daily in more than one in five Swedish men.^5^ This gives an opportunity to study the association between smoke‐free tobacco and reproductive function in a general population.

Three previous studies that have analysed the association between the use of moist oral snuff (from here on just mentioned as snuff) and male reproductive parameters have found somewhat different results. Richthoff et al. concluded that cigarette smoking, but not the use of snuff (which 51 men used) among young healthy men was associated with lower sperm counts.^6^ A similar result was found in Danish men (of whom 68 used snuff daily), in whom both daily cigarette and e‐cigarette users had decreased sperm counts.^7^ No association was found between snuff use and reproductive hormones in either of these two studies.

However, a study by Pärn et al. in men recruited from a fertility clinic (in which 17 men used snuff) has reported that men who used snuff had lower sperm counts and motility than the non‐users.^8^ Despite only one study reporting a deleterious association between snuff and semen quality, it is not known what possible substance related to the use of snuff that could have played a role. Still, nicotine has been suggested to have a harmful effect, since a study in rats reported impaired sperm parameters after treatment with nicotine.^9^ Another rat study showed a dose–response relationship between nicotine and impaired sperm parameters and fertility, and that ceasing nicotine treatment improved the sperm parameters.^10^

The amount of nicotine a human absorbs has been reported to be 1–1.5 mg/cigarette^11^ and 3.6 mg/portion of oral snuff.^12^ Snuff without tobacco has been reported to give a similar nicotine absorption to snuff (or snus) that contains tobacco.^13^ To examine nicotine exposure, levels of cotinine in biological fluids can be measured as a reliable marker.^14^

The aim of this study was to, with a larger number of users of snuff than in previous studies, investigate whether an association between the use of snuff and markers of reproductive function could be found, and if the weekly amount of snuff, or the extent of nicotine exposure, mattered.

## 2 Methods

### 2.1 Study design

This was an observational analytic study that determined the outcomes at the same time as the exposure, which is to be regarded as a cross‐sectional study.^15^

#### 2.1.1 Subjects

The first cohort was recruited 2000–2001 by Richthoff et al.^6^ Out of 2255 men who underwent a medical health examination at the National Service Administration in Sweden (NSAS) for a possible military service, 305 men chose to participate in the study. The participants were 18–21 years old and answered questions on smoking habits, long‐term/chronic diseases, and their time of abstinence. Since the information on the use of snuff was lacking, these men were contacted afterwards by telephone, and 51 out of the 242 men that were reached stated that they used snuff.^16^

The second cohort was recruited 2008–2010.^17^ Out of the 1618 men who underwent the medical health examination at NSAS during 2008–2010, 241 men chose to participate. Due to savings in the military budget, and thus a decrease in men going through the examination at NSAS, and to reach a sample size similar to the one in Richthoff et al., additionally 73 men in the age of 17–20 were recruited from high schools. The participants were asked to have a time of abstinence of 48–72 h. Prior to sampling and examination, the participants were asked to fill in a questionnaire regarding the time of abstinence, long‐term/chronic diseases, alcohol consumed (recalculated to the number of standard glasses of alcohol according to a Swedish system^18^) latest week, whether they used snuff [yes/no], how many boxes of snuff they consumed per week, if they were smoking, whether they smoked anything else than cigarettes, maternal occupation during the pregnancy, as well as regarding maternal and paternal smoking during the pregnancy.

All men from both cohorts were examined by a physician regarding a presence of varicocele, paid 500 SEK (about 55 Euro), delivered samples of urine and semen at the same day, and signed an informed consent. Body mass index (BMI) in both cohorts was assessed by the men's weight and length.

When combining data from the two cohorts, 619 participants were available for the study. Three participants in the earlier cohort and three in the later cohort answered that they smoked other products than cigarettes, and were excluded due to difficulty of classification whether they were smoker or not (for the later adjustment). After this exclusion, the study comprised 613 participants. Out of these 613 men, we had data in 538 men on whether they used snuff or not.

Data on the weekly consumed number of boxes of snuff were only collected in the later cohort, and these data were therefore only available in 71 of the snuff‐users. All men who reported not to use snuff were given the value of zero weekly boxes of snuff consumed, giving data on the numbers of boxes consumed per week in totally 493 participants.

The data collection and study of associations between exposure and reproductive function were approved (approval number 181/2008) by the local ethical review board at Lund University, and was in line with the Declaration of Helsinki. Regarding ethnicity, 73% of the men initially recruited during 2000–2001 were born and raised in Sweden and had mothers born and raised in Sweden, whereas only men fulfilling these two criteria were asked to participate in the recruitment that took place during 2008–2010.^19^

#### 2.1.2 Analysis of cotinine

Cotinine was analysed in the urine samples from 396 of the men, using liquid chromatography tandem mass spectrometry (LC–MS/MS; QTRAP5500, AB Sciex, Framingham, MA, USA) with a modified serum method^20^, ^21^ at the Division of Occupational and Environmental Medicine at Lund University. The limit of detection was 0.4 ng/ml, and the coefficient of variation at 48 ng/ml was 2%. The analysis of cotinine is part of a quality control program between analytical laboratories coordinated by the University of Erlangen‐Nuremberg, Germany.

#### 2.1.3 Semen quality

Sperm concentration (millions/ml), semen volume (ml), total sperm count (millions of sperms), percent morphologically normal sperms (%) and percent progressively motile sperms (%) were analysed according to the WHO guidelines used at the time.^22^ Furthermore, the sperm DNA fragmentation index was studied by use of the Sperm Chromatin Structure Assay,^23^ as previously briefly described for the two cohorts.^24^, ^25^

In addition, since the total number of progressively motile sperm (total progressively motile sperm count, TPMSC) has been suggested to be a better marker of fertility than using WHO cut‐off values with men below the fifth percentile considered abnormal,^25^ we calculated TPMSC number by multiplying the total sperm count with the proportion of progressively motile sperm.

### 2.2 Hormone levels

Levels of testosterone, luteinising hormone (LH), follicle‐stimulating hormone (FSH) and sex‐hormone binding globulin (SHBG) that were available from both cohorts, were measured in the second cohort by use of ElectroChemiLuminiscenceImmunoassay (Roche Cobas) as previously described in detail.^26^ In the first cohort, recruited around year 2000, these hormones were measured by use of an automated fluorescence detection system (Autodelfia®; Wallac Oy, Turku, Finland), which also has been detailed previously.^25^ Free testosterone was calculated according to a published method.^27^ In the first of the cohorts, we had data also on levels of inhibin B measured by an immunometric assay as described,^25^ whereas we in the second cohort had levels of oestradiol measured by an immunofluorometric method (Delfia Perkin‐Elmer).^26^

#### 2.2.1 Statistical methods

The analyses were executed in SPSS. The normal distribution of the outcome variables was examined. Total sperm count, sperm concentration, semen volume, DFI, percent progressively motile sperms and TPMSC were skewed. Since logarithmic or cubic root transformation have been suggested to best give the normal distribution for semen parameters,^28^ the skewed variables were transformed by their cubic roots, after which total sperm count, sperm concentration, TPMSC and semen volume showed normal distributions. Since DFI remained skewed, it was instead transformed by the natural logarithm to obtain a normal distribution. Percent progressively motile sperms did not show a normal distribution after transformation and was therefore used untransformed.

General linear models were used for all analyses. The analyses were adjusted for both cigarette smoking [yes/no] and the time of abstinence (missing in 18 men), since they both can influence semen quality.^28^ From the model, mean values were received, and Univariate Analysis of Variance (ANOVA) was performed to analyse the relationship of the binary variable [yes/no] with the semen parameters. Since the number of boxes of snuff consumed per week and cotinine levels were continuous variables, regression coefficients (*B*‐values) were received. After analysing the association between the use of snuff [yes/no] and the semen parameters, the mean values of sperm count, sperm concentration, semen volume and DFI in users and non‐users were back‐transformed to the original scale.

Finally, we also studied differences in levels of the reproductive hormones between the users and non‐users of snuff, as well as how cotinine levels and boxes of snuff consumed were associated with the reproductive hormones, adjusting for smoking (yes/no) and cohort number (1 or 2), since methods of chemical analyses differed between the two different cohorts.

Statistical significance level was set at 5%, getting two‐sided *p*‐values from SPSS.^29^ For statistically significant findings, to try to reduce the possibility of confounding, we adjusted separately also for either (1) long‐term chronic diseases (26 men yes, 491 men no, missing in 6 men); (2) varicocele (only available for the last recruited cohort; 26 yes, 267 no); (3) alcohol use latest week (available in 290 men [all from the last recruited cohort] with a mean of 5.8 glasses consumed); (4) maternal occupational field (classified using an internet‐based search function for occupation^30^ as 1: legislators, senior officials and managers [*n* = 4]; 2: professionals [*n* = 75]; 3: technicians and associate professionals [*n* = 29]; 4: clerks [*n* = 17]; 5: service workers and shop and market sales workers [*n* = 62]; 6: skill agricultural and fishery workers [*n* = 1]; 7: craft and related workers [*n* = 2]; 8: plant and machine operators and assemblers [*n* = 6]; and 9: elementary occupations [*n* = 3]);^31^ and (4) maternal and (5) paternal smoking during the pregnancy (maternal: 57 yes, 247 no, 309 missing; paternal: 87 yes, 208 no, 318 missing) all one at a time, all of which may also be associated with a decrease in semen quality as well as the use of snuff. These additional variables were added as either fixed factors or as a covariate (for alcohol use). A short comparison of the investigated potential confounders (except maternal occupational field) between the users and non‐users of snuff can be found in Table 1.

**TABLE 1:** Comparison of potential confounders between the users and non‐users of snuff

	Snuff users (*n* = 112)		Non‐users (*n* = 426)	
	*N*	Mean	*N*	Mean
Smoking (yes)	34	30%	85	20%
Abstinence time (h)	109	63	414	75
BMI	112	23	425	23
Varicocele (yes)	24	7.7%	217	10%
Long‐term/chronic disease (yes)	9	8.1%	19	4.5%
Maternal smoking (yes)	18	30%	38	16%
Paternal smoking (yes)	19	32%	64	28%
Standard glasses of alcohol latest week	65	7.9	237	5.2

[Correction added on 1 July 2022, after first online publication: The number and proportion of Snuff users and Non‐users for Smoking have been interchanged.]

## 3 RESULTS

### 3.1 Cotinine levels in urine

The mean levels of cotinine were 1100 ng/ml, with standard deviation (SD) 1700 ng/ml. The mean level in the 69 users of snuff was 3200 ng/ml (SD 2300 ng/ml), whereas the mean level in the 290 non‐users was 590 ng/ml (SD 1100 ng/ml).

### 3.2 Reproductive parameters in users of snuff versus non‐users of snuff

The men using snuff had 34 million or 24% lower total sperm count than the non‐users (*p* = 0.03, Table 2). Adding any of the additional separate adjustments (long‐term/chronic diseases, BMI, varicocele, the number of standard glasses of alcohol consumed last week, maternal occupational field, maternal or paternal smoking to the model) gave less than a 5% lowering of the estimate (the difference in total sperm count between the snuff users and non‐users).

**TABLE 2:** Comparison of reproductive parameters in men who used and did not use snuff, adjusted for smoking and abstinence time (semen parameters), or smoking and cohort (hormones)

	**Do use snuff**		**Do not use snuff**	
	***N***	**Mean (SD)**	***n***	**Mean (SD)**	***P* value**
Sperm concentration (×10^6^/ml)	101	47*	355	57*	0.10
Semen volume (mL)	101	2.8*	358	2.6*	0.33
Total sperm count (×10^6^)	109	120*	411	160*	0.03
TPMSC (×10^6^)	101	67*	356	84*	0.11
DFI (%)	101	11*	385	12*	0.32
Progressively motile (%)	100	42 (21)	358	42 (25)	0.98
Morphologically normal (%)	63	8.3 (5.8)	230	8.7 (6.9)	0.63
S‐Testosterone (nmol/L)	112	24 (4.8)	425	21 (16)	< 0.001
S‐Free testosterone (nmol/L)	112	0.43 (0.11)	425	0.43 (1.2)	0.81
S‐FSH (IU/L)	112	5.9 (7.8)	425	4.3 (9.1)	0.04
S‐LH (IU/L)	112	7.1 (9.3)	425	6.5 (11)	0.59
S‐SHBG (nmol/L)	112	31 (11)	425	31 (13)	0.98
S‐Oestradiol (pmol/L)	65	92 (24)	241	88 (29)	0.19
S‐Inhibin B (ng/L)	47	210 (63)	184	210 (71)	0.65

* Back‐transformed from the cubic root, whereas SD could not be back‐transformed to the original scale^41^

*Abbrevations*: DFI, DNA fragmentation index; *n*, number of men with available data; TPMSC, total progressively motile sperm count.

Regarding hormones, men who used snuff had 3.0 nmol/L higher levels of testosterone than the non‐users (*p* < 0.001), and 1.7 IU/L higher levels of FSH (*p* = 0.04, Table 2). None of the separate additional adjustments led to estimates (differences between the smokers and non‐smokers) that were lowered more than 1.1%.

### 3.3 Association between the numbers of boxes of snuff consumed per week and reproductive parameters

No statistically significant association was found between the number of boxes of snuff consumed per week and the semen parameters. However, such an association was found with both testosterone and FSH (both positive and with *p* < 0.001, Table 3). The separate additional adjustments gave less than a 3% lowering of the regression coefficients.

**TABLE 3:** Association between numbers of boxes of snuff used per week and reproductive parameters, adjusted for smoking, and abstinence time (semen parameters) or cohort (hormones)

	*N*	*B*	95% CI	*P* value
Sperm concentration (×10^6^/ml)^a^	417	–0.081	–0.20 to 0.04	0.17
Semen volume (ml)^a^	420	–0.015	–0.038 to 0.008	0.19
Total sperm count (×10^6^)^a^	473	–0.16	–0.33 to 0.014	0.07
TPMSC (×10^6^)^a^	418	–0.13	–0.30 to 0.04	0.13
DFI (%)^b^	444	–0.048	–0.11 to 0.018	0.15
Progressively motile (%)	444	–0.048	–0.11 to 0.018	0.15
Morfologically normal (%)	292	–0.25	–0.82 to 0.32	0.39
S‐Testosterone (nmol/L)	489	2.0	1.2 to 2.7	< 0.001
S‐Free testosterone (nmol/L)	489	–0.003	–0.013 to 0.007	0.52
S‐FSH (IU/L)	489	2.2	1.5 to 3.0	< 0.001
S‐LH (IU/L)	489	–0.07	–0.98 to 0.84	0.88
S‐SHBG (nmol/L)	489	–0.67	–1.7 to 0.33	0.19
S‐Oestradiol (pmol/L)	305	2.1	–0.17 to 4.3	0.07

^a^ Transformed by the cubic root.

^b^ Ln‐transformed.

Abbreviations: B, regression coefficient; DFI, DNA fragmentation index; *n*, number of men with available data; TPMSC, total progressively motile sperm count.

### 3.4 Association between the levels of cotinine and reproductive parameters

Levels of cotinine were negatively associated with the total sperm count (*p* = 0.03, Table 4). Additional adjustment for either long‐term/chronic diseases, BMI, varicocele, alcohol consumption or paternal smoking gave a maximum of 5% difference in the regression coefficients. When instead adjusting for maternal occupational field, this changed the coefficient 36% versus zero, and for maternal smoking 38% in the same direction.

**TABLE 4:** Correlation between the measured concentrations of cotinine in urine and sperm parameters (adjusted for smoking and abstinence time) and reproductive hormones (adjusted for smoking and cohort)

	*N*	*B*	95% CI	*P* value
Sperm concentration (× 10^6^ /ml)^a^	378	−0.032 × 10^−3^	−0.10 × 10^−3^ to 0.040 × 10^−3^	0.38
Semen volume (ml)^a^	316	−8.5×10^−5^	−1.9×10^−4^ to 1.7×10^−5^	0.10
Total sperm count (× 10^6^)^a^	345	−0.12 × 10^−3^	−0.23 × 10^−3^ to −0.01 × 10^−3^	0.02
TPMSC (× 10^6^)^a^	345	−8.9×10^−5^	−2.0×10^−4^ to 1.9×10^−5^	0.11
DFI (%)^b^	377	−0.022 × 10^−3^	−0.062 × 10^−3^ to 0.017 × 10^−3^	0.26
Progressively motile (%)	379	0.12 × 10^−3^	−0.98 × 10^−3^ to 0.012 × 10^−3^	0.83
Morphologically normal (%)	269	−0.081 × 10^−3^	−0.50 × 10^−3^ to 0.33 × 10^−3^	0.70
S‐Testosterone (nmol/L)	395	0.001	0.001 to 0.002	< 0.001
S‐FSH (IU/L)	395	0.0002	−0.0003 to 0.0008	0.38
S‐LH (IU/L)	395	−9.1×10^−5^	−0.001 to 0.001	0.78
S‐SHBG (nmol/L)	395	1.7×10^−5^	−0.001 to 0.001	0.96
S‐Oestradiol (pmol/L)	252	0.003	0.001 to 0.004	<0.001

^a^ Transformed by the cubic root.

^b^ Ln‐transformed.

Abbreviations: B, regression coefficient; DFI, DNA fragmentation index; *n*, number of men with available data; TPMSC, total progressively motile sperm count.

Regarding hormones, the levels of cotinine were positively associated with testosterone and oestradiol (*p* < 0.001 for both, Table 4), with the additional separate adjustments leading to a maximum of 5% decreased value of the regression coefficients.

## 4 DISCUSSION

We found that men who used smokeless tobacco in the form of snuff had 24% fewer sperm cells per ejaculation than men who did not use snuff (*P* = 0.03), and that cotinine levels in urine were positively associated with serum levels of testosterone and oestradiol (*P* < 0.001). Further, the number of boxes of snuff consumed per week was positively associated with levels of testosterone and FSH. All of these findings seemed robust to additional adjustment for use of alcohol, long‐term/chronic diseases, varicocele, maternal occupational group, and maternal and paternal smoking during the pregnancy.

A negative association between cotinine and total sperm count (*P* = 0.03) was, however, less robust to additional adjustment for either maternal occupational field or maternal smoking during the time of the pregnancy.

Previous studies are few in number and have differed in results. In the study published by Richthoff et al. on 242 men of which 51 used snuff, no association was found between the use of snuff and semen quality or reproductive hormones,^6^ whereas Pärn et al. reported an association between the use of snuff and lower total sperm count, sperm concentration, concentration of motile sperm and total motile sperm count.^8^ They used a sample of only 62 men (out of whom 17 were snuff users) but could still see a statistically significant difference in more variables than we did. This could possibly be explained by us using data from young healthy men, while Pärn et al. recruited their study participants through an infertility clinic. Thus, their participants may have had a higher probability of diverging sperm parameters regardless of the use of snuff, which in turn could make the association overestimated if applied to the general population. A later study including 68 men using snuff on a daily basis found no association with semen quality or reproductive hormones, albeit a lower total sperm count for daily users of cigarettes and e‐cigarettes.^7^ Few reviews on tobacco use and reproductive hormones seem to be performed, but smoking men were reported to have higher levels of testosterone than non‐smokers,^32^ and also oestradiol has been (dose‐relatedly) associated with men's smoking.^33^ However, a Danish study on about 3500 men found no association between men's smoking and oestradiol and FSH,^34^ which may argue for our finding on especially FSH to be spurious.

Regarding the fact that the association found between cotinine levels in urine and lower sperm count was attenuated by about a third when adjusting for maternal occupational field or maternal smoking may argue for the negative association between nicotine exposure and lower sperm count that we found, to be dependent on something else or additional to nicotine exposure through own use. However, in a post hoc analysis, only including users of snuff, but otherwise using the model described above, we could not see an attenuation of a negative regression coefficient between cotinine and total sperm count when adjusting for maternal occupational group, but a 40% reduction in the value of the coefficient when adjusting for maternal smoking. Taken together, our findings may argue for maternal smoking during pregnancy, potentially still occurring during the recruitment, as partly explaining the specific association between cotinine and lower total sperm count,^35^ whereas the lower total sperm count (and other findings) in the users of snuff than in the non‐users seemed independent on any of the separate additional potential confounders, including maternal smoking and maternal occupational group as an indicator of socioeconomic status. We found a non‐statistically significant negative association between boxes of snus consumed per week and total sperm count (*p* = 0.07) with the use of a two‐tailed *p*‐value, whereas one‐tailed *p*‐values (which are half the value of two‐tailed ones) have been suggested to be used when associations in a specific direction are anticipated, such as in the case of this study.^36^ As such, these results seem to be in line with snuff use to be associated with a lower total sperm count, potentially dose‐relatedly.

Cotinine is not a perfect marker of exposure, due to inter‐individual variations in metabolism.^14^ Accordingly, a self‐report of being a snuff user may potentially be a more stable marker of using snuff (and possibly also long‐term nicotine exposure) than a one‐time measurement of cotinine. Further, animal studies have reported that nicotine affected semen parameters in rats, including their sperm counts.^9^,^10^ Thus, together with the fact that almost two‐thirds of the value of the negative regression coefficient between cotinine and total sperm count remained after adjusting for the maternal smoking and occupational field, this might still indicate that the nicotine in snuff, at least to a certain extent could mediate a deterioration of semen quality in men exposed to nicotine via snuff, and in turn have implications also for tobacco‐free nicotine products. This would be in line with the lower total sperm counts reported in users of e‐cigarettes than in non‐users.^7^

Further studies on specifically tobacco‐free snuff would shed an additional light on whether nicotine could be a factor behind the lower sperm counts we found in snuff users. Moreover, it would be interesting to see studies investigating if sperm parameters normalise after a cessation of snuff use.

### 4.1 Strengths and limitations

In comparison to the articles by Pärn et al.,^8^ Richthoff et al.^16^ and Holmboe et al.,^7^ our article included a higher number of snuff‐users, and thus possibly had a greater statistical power. That may increase the likelihood of the results being representative of the population. The fact that the association between the use of snuff and total sperm count seemed to lie in the same direction as associations between the levels of cotinine in urine and total sperm count (as an additional way of evaluating the association between the use of snuff and semen quality) seems to strengthen the possibility of causality.

Weaknesses of our study include the fact that several variables were only available in part of the men, such as the numbers of boxes of snuff used per week, maternal occupational field, etc. This may have led to an underestimation of true associations.^37^ Furthermore, our adjustments for the maternal occupational field and maternal and paternal smoking may not have fully adjusted for socioeconomic status, which has been reported negatively associated with semen quality,^8^ having in mind that socioeconomic status does affect health outcomes via factors that include the use of tobacco.^38^ The fact that we found no statistically significant association between the numbers of boxes of snuff consumed per week and semen parameters might be due to the fact the time of keeping the oral snuff in the mouth may vary between different individuals, despite potentially consuming the same amount of boxes per week. Larger studies would be needed to determine a possible relationship between the extent of snuff consumed and semen parameters.

Finally, the design of this study does not allow to decide whether the associations we found were causal. Therefore, such possibilities must be seen in view of findings from experimental studies, which however speak in a similar direction regarding decreased sperm counts after nicotine exposure.^9^, ^10^ Nonetheless, we can still not exclude that other factors related to the use of snuff or exposure to nicotine may have been playing a role for the associations with sperm count and reproductive hormones in the men included in this study.

### 4.2 Interpretation 

Although we found no statistically significant association between the use of snuff and TPMSC, which is reported to be associated with spontaneous pregnancy,^25^ the lower total sperm count in men who used snuff (120×10^6^ vs. 160×10^6^) may be clinically relevant, despite being above the lower WHO reference limit,^39^ since a correlation has been reported between total sperm count and the chance of fathering a child up to a level of 200 million sperms.^40^ Each decrease of 10 million sperms below a total sperm count of 200 million was reported to be correlated with a subsequent 3% lower chance of fathering a child.^40^ With the difference we found of 34 million sperms between the groups, the chance of fathering a child for those who used snuff should thus equal 0.97^3^ ^.^ ^4^ times that of the non‐users. That would equal 9.8% decreased chance of fathering a child in the users of snuff, with a potential dose‐related association.

## 5 CONCLUSIONS

We found lower sperm counts in men using smoke‐free tobacco in the form of moist oral snuff, as compared with non‐users. Given previously reported associations between total sperm count and the chance of pregnancy, our findings may implicate an association between the use of smoke‐free tobacco and a lower chance of fathering a child.

## CONLICT OF INTEREST

The authors declare that there is no conflict of interest that could be perceived as prejudicing the impartiality of the research reported.

## AUTHOR CONTRIBUTIONS

Agnes Kimblad and Gustav Ollvik performed the statistical analyses and wrote the draft of the manuscript. Christian H. Lindh was responsible for the analysis of cotinine. Jonatan Axelsson provided data and revised the manuscript. All authors agreed to the final version of the manuscript.

[Correction added on 1 July 2022, after first online publication: The references have been renumbered after ref. [24].]
